# Commentary: Proteomics Analysis Revealed that Crosstalk between *Helicobacter pylori* and *Streptococcus mitis* May Enhance Bacterial Survival and Reduces Carcinogenesis

**DOI:** 10.3389/fmicb.2017.02381

**Published:** 2017-11-29

**Authors:** Paweł Krzyżek

**Affiliations:** Department of Microbiology, Wrocław Medical University, Wrocław, Poland

**Keywords:** *Helicobacter pylori*, Streptococcus, gastric cancer, stomach, virulence, coccoid bacteria, co-culture

For a long time, it was thought that the stomach is a sterile organ, free from the presence of microorganisms (Nardone and Compare, 2015). A breakthrough in thinking about stomach as a site possible for colonization was the discovery of Marshall and Warren (1984), who managed to isolate *Helicobacter pylori*, a spiral Gram-negative bacterium, from human gastric biopsies. Since then, many researchers have focused their attention on the gastric microbiota studies. It was determined that the amount of microorganisms inhabiting this niche counts of 10^2^–10^4^ CFU/ml and varies in different stomach regions. Firmicutes and Proteobacteria prevail within the gastric mucosa, while Firmicutes, Bacteroidetes, and Actinobacteria dominate in the gastric juice (Ianiro et al., 2015; Nardone and Compare, 2015). The presence of non-*H. pylori* microbes suggests the possibility that gastric microflora may interact with *H. pylori* and modulate physiology and morphology of this bacterium. Therefore, data from researches determining such dependencies are extremely valuable.

An example of one such relevant research was the analysis of *Streptococcus mitis*, a physiological inhabitant of the human gastrointestinal tract, and *H. pylori* co-culture effect on the proteomes of both bacteria (Khosravi et al., 2016). The idea to determine the impact of *S. mitis* on *H. pylori* physiology was derived from previous researchers' observations that *S. mitis* may induce morphological transformation of *H. pylori* from spiral to coccoid form (Khosravi et al., 2014). Co-culture of both bacteria significantly changed *H. pylori* proteomic profile, including the increase of proteins involved in DNA repair and genetic rearrangements, and the reduction of proteins responsible for metabolism and antioxidant activity (Khosravi et al., 2016). I believe that at this point Authors made a large mental jump, claiming that lower levels of antioxidant enzymes in *H. pylori* may be associated with a decrease in oxidative stress in the stomach tissue and subsequently lower risk of the cancer development. The conclusion of this study, as reflected in the title, was that *S. mitis* may contribute to a reduction in the *H. pylori*-induced carcinogenesis. The hypothesis although not necessarily incorrect, should be confirmed by experiments in later parts of this article, or be made as a minor suggestion in the Discussion section, giving the opportunity to verify this hypothesis (by a team of these or other researchers). In my opinion, it seems that: (1) the conditions in the intracellular environment (in this case *H. pylori*) are different from those in the extracellular environment (gastric mucosa niche), and there is no simple relation between conditions within both regions, (2) the decrease of proteins associated with *H. pylori* antioxidant activity does not necessarily have an adversely effect on the production of virulence factors inducing the gastric mucosa inflammation. Rejection or confirmation of the Khosravi et al. (2016) hypothesis can be made, for example, by the co-culture of both bacteria (*H. pylori* and *S. mitis*) in the presence of eukaryotic cell lines, physiological, and with induced inflammatory or neoplastic changes. Reduction of inflammation/carcinogenesis intensity in eukaryotic cells during *H. pylori* and *S. mitis* co-culture, in compared to *H. pylori* monoculture control, could provide evidence of Authors finding and open a new pathway in *in vitro* and *in vivo* studies.

The genus *Streptococcus* belong to microflora naturally occurring in the human body, especially in the digestive tract. For this reason, these bacteria are commonly used as probiotics, i.e., microorganisms with beneficial effects on the human body functions. This includes acting as an immunomodulatory, anti-inflammatory, and anti-allergic agent and protecting against gastrointestinal pathogens infection (including *H. pylori*) (Markowiak and Slizewska, 2017). For example, Engen et al. (2017) have shown that *S. mitis* cell lysate was capable of initiating wound healing by the aryl hydrocarbon receptor activation (AhR) and AhR-dependent leukocyte recruitment, involved in the remodeling of damaged tissues.

There are also studies showing the potential of *Streptococcus* to initiate pathogenic processes. Significantly more frequent isolation and increased numbers of these microorganisms, including *S. mitis*, have been observed in patients with antral gastritis (Li et al., 2009), as well as with gastric (Eun et al., 2014; Choi et al., 2017; Sohn et al., 2017), esophageal (Narikiyo et al., 2004; Chen et al., 2015), and oral squamous cancers (Mager et al., 2005). The relationship between the presence of these bacteria and the progression of the above-mentioned pathologies is not known. Both the possibility of stimulation of chemokines involved in the monocytes and neutrophils recruitment (Narikiyo et al., 2004) and the presence of very active alcohol dehydrogenase (ADH), which contributes to the high local concentrations of carcinogenic acetaldehyde (Kurkivuori et al., 2007), are suggested. One of the well-known examples of species from the genus *Streptococcus*, able to directly induce neoplasms, is *Streptococcus gallolyticus* spp. *gallolyticus*. The pro-carcinogenic potential of this bacterium includes the capacity of selective adhesion to tumor-altered tissue (colonic mucous), induction of increased cell proliferation, and activation of cytokines (IL-1, IL-8, IFN-γ) and transcriptional factors (NF-κB) associated with exacerbate inflammation (Abdulamir et al., 2011; Kumar et al., 2017).

The ability of *Streptococcus mitis* to promote gastric carcinogenesis, if any, does not need to be directly related to the virulence factors presence and the initiation of pathological changes by these microorganisms. Participation in the oncogenesis initiation may be indirect, consisting the induction of morphological transition of *H. pylori* into a coccoid form. This model is in agreement with the observations made by Authors in another study (Loke et al., 2016). *H. pylori* spherical forms have been shown to have a higher level of proteins associated with the intensification of carcinogenesis than spiral forms. Hence, *Streptococcus mitis* could even contribute *in vivo* to the enhancement of *H. pylori*-mediated gastric cancer development. This model is hypothetical and should be proven experimentally.

The statement presented in this commentary does not in any way diminish the relevance or value of the research conducted by Khosravi et al. (2016). On the contrary, it simply seeks to understand the complexity of bacteria-bacteria and host-bacteria interactions, and makes a suggestion for further research in this area.

## Author contributions

After having carefully read the recent Frontiers in Microbiology article by Yalda Khosravi and coworkers, upon which this manuscript is based, the Author (PK) has autonomously and independently written the present Commentary.

### Conflict of interest statement

The author declares that the research was conducted in the absence of any commercial or financial relationships that could be construed as a potential conflict of interest.
